# Predictive factors for pCR and relapse following neoadjuvant dual HER2-blockade in HER2+ breast cancer: an international cohort study

**DOI:** 10.1007/s12094-025-03937-7

**Published:** 2025-05-10

**Authors:** Paulo Luz, Raquel Lopes-Brás, Inês Soares de Pinho, Vanessa Patel, Miguel Esperança-Martins, Lisa Gonçalves, Joana Gonçalves, Rita Freitas, Diana Simão, Maria Roldán Galnares, Silvia Artacho Criado, Amanda Nobre, Elias A. Gracia Medina, Isabel M. Saffie Vega, Rita Teixeira de Sousa, Luís Costa, João Gregório, João G. Costa, Ana S. Fernandes

**Affiliations:** 1 CBIOS - Universidade Lusófona’s Research Center for Biosciences & Health Technologies, Lisbon, Portugal; 2 Departament of Biomedical Sciences, Universidad de Alcalá, Madrid, Spain; 3 Medical Oncology, Unidade Local de Saúde do Baixo Alentejo, Beja, Portugal; 4 Medical Oncology Department, Unidade Local de Saúde de Santa Maria, Lisbon, Portugal; 5 Medical Oncology Department, Unidade Local de Saúde do Arco Ribeirinho, Barreiro, Portugal; 6 Medical Oncology Department, Unidade Local de Saúde de Amadora/Sintra, Amadora, Portugal; 7 Medical Oncology Department, Unidade Local de Saúde de São José, Lisbon, Portugal; 8 Pharmacy Departament, Hospital Virgen de Valme, Seville, Spain; 9 Medical Oncology Department, Unidade Local de Saúde de Entre Douro e Vouga, Santa Maria da Feira, Portugal; 10 National Institute of Oncology and Radiobiology, La Habana, Cuba; 11 Reconstructive and Oncological Breast Surgery Unit, Arturo López Pérez Foundation, Providencia, Chile

**Keywords:** HER2 positive breast cancer, Anti-HER2 neoadjuvant therapy, Pathological complete response, Real-world evidence

## Abstract

**Purpose:**

Neoadjuvant systemic therapy with dual HER2-blockade, trastuzumab and pertuzumab, combined with chemotherapy has become a standard approach in patients with HER2-positive (HER2+) breast cancer (BC). However, the variability in treatment outcomes, such as pathological complete response (pCR) or relapse rates, underscores the need to identify predictive factors to optimize therapeutic strategies. This study aims to explore the relationship between clinicopathological factors and both pCR and disease-free survival (DFS) in an international cohort of patients with HER2+ BC, contributing to defining personalized treatment strategies.

**Methods:**

An international, multicenter, retrospective cohort study was conducted, including 517 patients with HER2+ BC who received neoadjuvant therapy comprising trastuzumab, pertuzumab, and chemotherapy. Data were collected between January 2016 and December 2023. The relationship between clinicopathological factors and treatment outcomes was analyzed using univariate tests, logistic regression for pCR, and Cox proportional hazards regression for DFS. Kaplan–Meier survival curves with log-rank tests and hazard ratios were used to compare DFS across subgroups.

**Results:**

Multivariable analysis revealed that hormonal receptor (HR) expression and nodal status significantly predicted the achievement of pCR in this cohort. Factors such as age, HR status, tumor grade, Ki-67 index, nodal status, and pathological response were associated with relapse risk.

**Conclusion:**

Our real-world data demonstrates that a comprehensive approach considering pCR, age, HR status, and nodal involvement is essential for personalized treatment strategies. These factors should be taken into account when deciding whether to escalate or de-escalate treatment, contributing to improved HER2+ BC patient outcomes.

**Graphical abstract:**

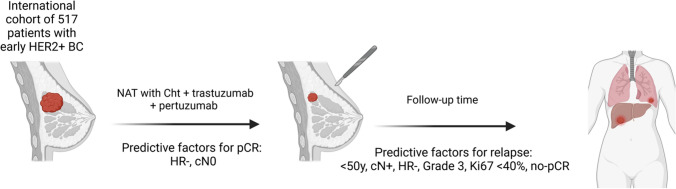

**Supplementary Information:**

The online version contains supplementary material available at 10.1007/s12094-025-03937-7.

## Introduction

In breast cancer (BC), particularly the HER2-positive (HER2+) subtype, neoadjuvant therapy (NAT) serves as a crucial platform for translational research, drug development, and biomarker evaluation [1]. A key endpoint in these studies is the achievement of complete pathological response (pCR), which is commonly associated with improved long-term outcomes, including disease-free survival (DFS).

There is an urgent need to deepen our understanding of the factors influencing both pCR and relapse in real-world settings. Real-world studies are essential in addressing these questions, as they offer insights into the performance of treatments in broader, more diverse patient populations outside the controlled environment of clinical trials. By reflecting the complexities of real-life clinical practice, these studies provide valuable data on treatment effectiveness, safety, and outcomes in a range of patient profiles [2].

Patients with HER2+ BC who achieve pCR after NAT typically have a favorable prognosis, with higher rates of DFS [3, 4]. However, despite pCR being a strong predictor of better outcomes, some patients, particularly those with high tumor burden (*e.g.* large tumors or significant lymph node involvement), still experience relapse [5]. Moreover, in hormone receptor (HR)-positive HER2+ BC, the correlation between pCR and DFS is less robust than in HR-negative cases, underscoring the complexity of the factors contributing to both pCR and relapse [3].

Currently, NAT with dual HER2 blockade using trastuzumab and pertuzumab is the standard treatment approach for HER2+ BC when the tumor size is ≥ 2 cm and/or nodal involvement is present. NAT is also recommended for smaller tumors (1–2 cm) if high-risk features, such as younger age, high-grade, or elevated Ki-67 index, are identified. This approach can also facilitate breast-conserving surgery in cases where mastectomy might otherwise be required [6].

Building on this foundation, the combination of trastuzumab and pertuzumab has been established as a superior neoadjuvant treatment strategy for HER2+ BC. The NeoSphere trial demonstrated that adding pertuzumab to trastuzumab and docetaxel significantly increased the pCR rate compared to trastuzumab and docetaxel alone (45.8% vs. 29.0%) [7]. This dual blockade approach has been integrated into clinical guidelines, reflecting its enhanced efficacy [6].

Concerns about the cardiotoxicity associated with anthracyclines have prompted investigations into their necessity in neoadjuvant regimens. The TRAIN-2 trial compared anthracycline-containing chemotherapy with anthracycline-free regimens, both combined with dual HER2 blockade. The study found no significant difference in pCR rates between the two groups, suggesting that anthracycline-free regimens could be a viable option, potentially reducing treatment-related toxicity [8].

Achieving a pCR after neoadjuvant therapy is associated with improved long-term outcomes. A pooled analysis by the FDA, which included 11,955 patients across 12 neoadjuvant trials, demonstrated that pCR was correlated with favorable event-free survival (EFS) and overall survival (OS), particularly in HER2+, hormone receptor-negative BC [3]. This underscores the importance of pCR as a surrogate endpoint in clinical trials and its prognostic significance in clinical practice.

Patients who achieve pCR with neoadjuvant treatment experience a significantly improved prognosis, with a 5-year DFS rate of around 85%, compared to 76% in those who do not achieve pCR [3–5]. For patients who do not achieve pCR, the risk of relapse remains a concern. The KATHERINE trial addressed this by evaluating the efficacy of T-DM1 in patients with residual invasive disease after neoadjuvant therapy. Adjuvant T-DM1 significantly reduced the risk of invasive disease recurrence or death compared to trastuzumab alone, leading to its adoption as the standard adjuvant therapy in this high-risk population [9]. In HR-positive HER2+ BC, optimizing endocrine therapy is also another key factor influencing outcomes [6].

This study investigated the relationship between clinicopathological factors, pCR, and relapse in HER2+ BC patients treated with NAT, incorporating chemotherapy, trastuzumab, and pertuzumab, in a real-world context, using an international cohort. Identifying these critical factors could help pinpoint patients who may not fully benefit from current therapies, guiding the development of alternative strategies (e.g., clinical trials) and supporting the selection of candidates for treatment de-escalation approaches.

## Material and methods

This research is supported by an international, multicenter, retrospective cohort study designed to explore the association of several biomarkers with treatment outcomes of patients with HER2+ BC. Patients’ data were collected from multiple hospitals between January 2016 and December 2023.

### Dataset

The study utilised secondary data obtained from the electronic medical records of each participating hospital (n = 9): in Portugal (6), Spain (1), Chile (1), and Cuba (1). Patients were selected according to the databases maintained by each hospital, which record treated patients at their respective centers. Patients included in this study were those diagnosed with early or locally advanced HER2+ BC (Stage I–III) who received a regimen of NAT comprising chemotherapy, trastuzumab and pertuzumab, followed by surgical intervention. Eligible patients received doxorubicin/epirubicin and cyclophosphamide for 4 cycles, followed by weekly paclitaxel for 12 weeks, trastuzumab, and pertuzumab every 21 days for 4 cycles, or alternatively, TCHP (docetaxel, carboplatin, trastuzumab, and pertuzumab) every 21 days for 6 cycles. To ensure adequate exposure to anti-HER2 therapy, only patients who received at least two cycles of trastuzumab and pertuzumab were included. The index date, which served as the baseline for this study, was defined as the date of diagnosis of HER2+ BC. The period for inclusion spanned from January 2016 to December 2023.

To ensure patient confidentiality, data were transferred in a pseudoanonymized manner into an electronic case report form. Prior to data collection, ethical approval was obtained from the Ethics Committee of the School of Health Sciences and Technologies (Ref. CE.ECTS/P23-23), as well as from each site's respective board to ensure compliance with national and international guidelines for research involving human subjects. Patients received treatments based on local prescribing information and routine medical practices, and only data from these routine practices were collected for the study.

### Study variables

Patient characteristics were collected through the retrospective analysis of clinical records by the principal investigator of each center. These variables included age at diagnosis, tumor size (cT), clinical nodal status (cN), histological subtype, tumor grade, and immunohistochemical evaluations of HR, HER2 status, and Ki67. Additionally, details regarding the neoadjuvant chemotherapy regimen, type of surgery performed, adjuvant treatments, pathological response, and the dates of relapse and local recurrence were collected. pCR was specifically defined as the absence of invasive cancer in the breast and axillary nodes (ypT0/is ypN0). DFS was defined as the interval from the date of disease diagnosis to any of the events: recurrence of ipsilateral invasive or non-invasive BC, recurrence of ipsilateral locoregional invasive BC, contralateral invasive BC (excluding contralateral in situ disease), or distant disease recurrence.

### Statistical analysis

Descriptive statistics were used to summarise patient characteristics and other relevant study variables. The normality of the continuous variables was assessed using the Shapiro–Wilk test, ensuring the appropriate statistical methods were applied based on the distribution of the data. Continuous variables were described using means and standard deviations, or medians and interquartile ranges (IQRs) as appropriate. Ordinal and categorical variables were summarized using frequencies and percentages.

Missing data were addressed by excluding variables with more than 25% missing values. This threshold was defined to reduce the risk of bias and ensure the robustness of the analysis. All patients were retained in the dataset, and no imputation methods were applied. Only complete cases were used for each analysis, meaning that patients were included in the analysis of a given variable only if they had complete data for that specific variable. This approach allowed us to preserve the overall sample size while maintaining the integrity of the results. In total, 2 variables were excluded (smoking habits and body mass index) due to exceeding the 25% missing data threshold. The final analysis included 15 variables with acceptable completeness. The distribution of patients and treatments received is detailed in Table 1.

**Table 1:** Patient characteristics per country

Patient characteristics	Total (N = 517)	Portugal (N = 292)	Spain (N = 47)	Chile (N = 89)	Cuba (N = 89)	*p* value
Median age, years (Q1; Q3)	52.8 (45.0; 61.0)	52.4 (45.1; 61.4)	47.5 (41.6; 55.8)	53.0 (45.0; 62.0)	54.9 (48.8; 59.8)	0.127
Menopausal status						
Pre-menopausal, n (%)	173 (40.7)	116 (40.1)	28 (59.6)	–	29 (32.6)	**0.010**
Peri/post-menopausal, n (%)	252 (59.3)	173 (59.9)	19 (40.4)	–	60 (67.4)	
HR						
Negative, n (%)	181 (35.0)	81 (27.7)	26 (55.3)	34 (38.2)	40 (44.9)	**< 0.001**
Positive, n (%)	336 (65.0)	211 (72.3)	21 (44.7)	55 (61.8)	49 (55.1)	
Ki-67%, median (Q1, Q3)	40.0 (25.0; 57.3)	40.0 (25.0; 60.0)	40.0 (30.0; 60.0)	–	30.0 (20.0; 50.0)	**0.007**
Tumor grade				–		
Grade 1–2, n (%)	208 (51.1)	163 (56.2)	12 (37.5)	–	33 (38.8)	**0.005**
Grade 3, n (%)	199 (48.9)	127 (43.8)	20 (62.5)	–	52 (61.2)	
cT						
T1–2, n (%)	344 (66.5)	203 (69.5)	22 (46.8)	65 (73.0)	54 (60.7)	**0.006**
T3–4, n (%)	173 (33.5)	89 (30.5)	25 (53.2)	24 (27.0)	35 (39.3)	
cN						
N0, n (%)	221 (42.7)	147 (50.3)	13 (27.7)	43 (48.3)	18 (20.2)	** < 0.001**
N+, n (%)	296 (57.3)	145 (49.7)	34 (72.3)	46 (51.7)	71 (79.8)	
Neoadjuvant chemotherapy						
Anthracycline-based, n (%)	354 (68.5)	272 (93.2)	16 (34.0)	4 (4.5)	62 (69.7)	** < 0.001**
TCHP, n (%)	163 (31.5)	20 (6.8)	31 (66.0)	85 (95.5)	27 (30.3)	
Adjuvant anti-HER2						
Trastuzumab, n (%)	435 (84.1)	249 (85.3)	40 (85.1)	69 (77.5)	77 (86.5)	0.064
Trastuzumab + Pertuzumab, n (%)	13 (2.5)	11 (3.8)	2 (4.3)	0 (0.0)	0 (0.0)	
T-DM1, n (%)	65 (12.6)	29 (9.9)	5 (10.6)	19 (21.3)	12 (13.5)	
None, n (%)	4 (0.8)	3 (1)	0 (0.0)	1 (1.1)	0 (0.0)	
Adjuvant endocrine therapy						
Tamoxifen, n (%)	68 (20.2)	49 (23.2)	8 (38.1)	0 (0.0)	11 (22.4)	**< 0.001**
AI, n (%)	167 (49.7)	99 (54.7)	6 (28.6)	34 (61.8)	28 (57.1)	
Tamoxifen + OFS, n (%)	30 (8.9)	24 (11.4)	2 (9.5)	1 (1.8)	3 (6.1)	
AI + OFS, n (%)	26 (7.7)	22 (10.4)	1 (4.8)	3 (5.5)	0 (0.0)	
Unknown, n (%)	45 (13.4)	17 (0.8)	4 (19.0)	17 (30.9)	7 (14.3)	
Type of primary surgery						
Mastectomy, n (%)	274 (53.0)	132 (45.2)	25 (53.2)	43 (48.3)	73 (83.9)	**< 0.001**
BCS, n (%)	243 (47.0)	160 (54.8)	22 (46.8)	46 (51.7)	14 (16.1)	
Type of axillary surgery						
Complete axillary dissection, n (%)	268 (51.8)	129 (44.2)	30 (63.8)	25 (28.1)	84 (94.4)	**< 0.001**
SLN, n (%)	242 (46.8)	157 (53.8)	17 (36.2)	64 (71.9)	4 (4.5)	
None, n (%)	7 (1.4)	6 (2.1)	0 (0.0)	0 (0.0)	1 (1.9)	
Adjuvant radiotherapy	253 (48.9)	162 (55.5)	23 (48.9)	48 (53.9)	20 (22.5)	0.457
Treatment outcome						
pCR, n (%)	315 (60.9)	173 (59.2)	26 (55.3)	61 (68.5)	55 (61.8)	0.367
Relapse, n (%)	39 (7.5)	20 (6.8)	4 (8.5)	4 (4.5)	11 (12.5)	0.220

*AI* aromatase inhibitors, *BCS* breast conservative surgery, *cT* tumor size, *cN* lymph node disease, *HR* hormonal receptor, *OFS* ovary function suppression, *pCR* pathologic complete response, *TCHP* docetaxel, carboplatin, trastuzumab and pertuzumab, *SLN* sentinel lymph node biopsy

Bold values indicate statistical significance at *p* < 0.05

For statistical inference, continuous variables were compared using Student’s t-test or Mann–Whitney U test, depending on the data distribution. When comparing more than two groups, one-way ANOVA or Kruskal–Wallis tests were applied. Categorical variables were compared using Chi-squared or Fisher's exact tests to identify predictors of pCR and relapse. Variables with p < 0.05 in univariate analysis were included in Multivariate Model 1. A second model (Model 2) was also constructed, including additional clinically relevant variables, regardless of their univariate significance, to account for potential confounding factors and explore robustness. DFS was conducted using Kaplan–Meier methods to estimate the survival functions and visualize time-to-event data. The Kaplan–Meier curves were compared using the log-rank test to assess differences between groups. Additionally, the hazard ratio was obtained using Cox proportional hazards regression analysis, which models the time to an event (such as relapse) and accounts for the effects of multiple covariates.

Statistical significance was established at a p-value of less than 0.05. Additionally, hazard ratio and 95% confidence intervals (CI) were calculated. All statistical tests were two-sided, and analyses were executed using Jamovi software version 2.3.28 [10].

## Results

### Clinical and pathological characteristics of enrolled patients

A total of 517 patients with HER2+ BC who received neoadjuvant trastuzumab, pertuzumab, and chemotherapy were included in this study. The baseline characteristics of the patients are presented in Table 1.

The median age of the patients was 52.8 years. Of the total, 252 patients (59.3%) were peri- or post-menopausal, while 173 patients (40.7%) were pre-menopausal. Regarding HR status, 336 patients (65%) had HR-positive tumors, and 181 patients (35%) had HR-negative tumors. The median Ki67 in the biopsy available for 507 patients, was 40%. Histologic grading was reported for 405 patients, with 208 (51.1%) having grade 1–2 tumors. At diagnosis, 344 patients (66.5%) had tumors classified as T1-2, while 173 patients (33.5%) had tumors classified as T3-4. Lymph node involvement was negative (N0) in 221 patients (42.7%) and positive (N+) in 296 patients (57.3%).

NAT was anthracycline-based in 354 patients (68.5%) and TCHP in 163 patients (31.5%). For adjuvant anti-HER2 therapy, 435 patients (84.1%) received trastuzumab, 13 patients (2.5%) received both trastuzumab and pertuzumab, 65 patients (12.6%) received T-DM1, and 4 patients (0.8%) received no adjuvant anti-HER2 therapy (Supplementary Table 1). For those patients with HR-positive tumor, adjuvant endocrine therapy was administered as follows: 68 patients (20.2%) received tamoxifen, 167 patients (49.7%) received aromatase inhibitors (AI), 30 patients (8.9%) received tamoxifen with ovarian function suppression (OFS), and 26 patients (7.7%) received AI with OFS.

Regarding surgical treatment, 274 patients (53%) underwent mastectomy, while 243 patients (47%) had breast-conserving surgery (BCS). Axillary surgery included complete axillary dissection in 268 patients (51.8%), sentinel lymph node biopsy (SLNB) in 242 patients (46.8%), and no axillary surgery in 7 patients (1.4%). pCR was achieved in 315 patients (60.9%), and 39 patients (7.5%) experienced a relapse. The median follow-up time was 2.98 years.

### Predictive factors of pCR

In the univariate analysis (Supplementary Table 2), HR status emerged as a significant predictor of achieving pCR in HER2+ BC patients (OR 0.371, 95% CI 0.253–0.562). Multivariate analysis (Table 2) further confirmed HR status as an independent predictor of pCR (OR 0.459, 95% CI 0.289–0.728), along with nodal status (OR 0.587, 95% CI 0.379–0.907).

**Table 2:** Univariate and multivariate analysis of predictive factors for pathological complete response (pCR) in patients with HER2+ breast cancer

Predictor	Univariate model, OR (IC 95%)	Multivariate model, OR (IC 95%)
Age (≥ 50 vs < 50)	0.993 (0.693–1.420)	0.882 (0.581–1.340)
cT (T3-T4 vs T1-T2)	0.950 (0.654–1.380)	0.954 (0.610–1.492)
cN (N+ vs N0)	0.757 (0.528–1.080)	**0.587 (0.379–0.907)**
HR (positive vs negative)	**0.371 (0.253–0.562)**	**0.459 (0.289–0.728)**
Grade (3 vs 1–2)	1.230 (0.831–1.830)	1.168 (0.759–1.796)
Ki-67 (≥ 40% vs < 40%)	1.150 (0.783–1.700)	1.017 (0.665–1.555)
Neoadjuvant anthracycline (yes vs no)	0.870 (0.593–1.280)	1.170 (0.678–2.020)

Multivariate analysis includes all variables listed, selected based on clinical relevance and literature support, regardless of their statistical significance in the univariate analysis. Bold values indicate statistical significance at *p* < 0.05

*cT* tumor size, *cN* lymph node disease, *HR* hormonal receptor

### Predictive factors of relapse

Univariate analysis revealed several significant factors impacting the risk of relapse among patients with HER2+ BC. These included age ≥ 50 years (OR 0.459, 95% CI 0.236–0.891), tumor size (T3–T4 vs T1–T2) (OR 2.236, 95% CI 1.160–4.312), cN status (N+ vs N0) (OR 3.115, 95% CI 1.402–6.917), and pCR (OR 0.329, 95% CI 0.167–0.650) (Supplementary Table 3).

Two multivariate models for simultaneous (*i.e.* enter method) logistic regressions were produced. Multivariate Model 1 including the aforementioned factors identified age ≥ 50 years (OR 0.417, 95% CI 0.101–0.826), cN status (N+ vs N0) (OR 2.716, 95% CI 1.773–6.266), and pCR (OR 0.335, 95% CI 0.168–0.671) as associated with relapse risk.

Subsequent multivariate analysis (Multivariate model 2) including relevant clinical-pathological factors affirmed the significance of age ≥ 50 years (OR 0.296, 95% CI 0.133–0.659), cN status (N+ vs N0) (OR 2.936, 95% CI 1.107–7.782), and pCR (OR 0.312, 95% CI 0.137–0.709) as independent predictors of relapse risk. Additionally, the HR status (positive vs negative) (OR 0.424, 95% CI 0.183–0.979) tumor grade (3 vs 1–2) (OR 2.661, 95% CI 1.134–6.242) and Ki-67 (≥ 40% vs < 40%) (OR 0.330, 95% CI 0.145–0.751), were also identified as significant independent factors that influence the risk of relapse (Table 3).

**Table 3:** Univariate and multivariate analysis of predictive factors for relapse in patients with HER2+ breast cancer

Predictor	Univariate Model, OR (IC 95%)	Multivariate Model 1, OR (IC 95%)	Multivariate Model 2, OR (IC 95%)
Age (≥ 50 vs < 50)	**0.459 (0.236–0.891)**	**0.417 (0.101–0.826)**	**0.296 (0.133–0.659)**
cT (T3-4 vs T1-2)	**2.236 (1.160–4.312)**	1.780 (0.8895–3.560)	1.154 (0.516–2.579)
cN (N+ vs N0)	**3.115 (1.402–6.917)**	**2.716 (1.773–6.266)**	**2.936 (1.107–7.782)**
HR (positive vs negative)	0.676 (0.349–1.308)	–	**0.424 (0.183–0.979)**
Grade (3 vs 1–2)	2.1129 (0.991–4.505)	–	**2.661 (1.134–6.242)**
Ki-67 (≥ 40% vs < 40%)	0.685 (0.342–1.372)	-	**0.330 (0.145–0.751)**
Neoadjuvant anthracycline (yes vs no)	1.5844 (0.734–3.419)	–	2.359 (0.729–7.635)
pCR (yes vs no)	**0.329 (0.167–0.650)**	**0.335 (0.168–0.671)**	**0.312 (0.137–0.709)**

Multivariate Model 1 includes only variables that were statistically significant in univariate analysis (*p* < 0.05). Multivariate Model 2 includes both statistically significant variables and additional clinically relevant variables based on literature and biological plausibility. Bold values indicate statistical significance at *p* < 0.05

*cN* lymph node disease, *HR* hormonal receptor, *cT* tumor size, *pCR* pathological complete response

### Survival analysis

**Fig. 1:**
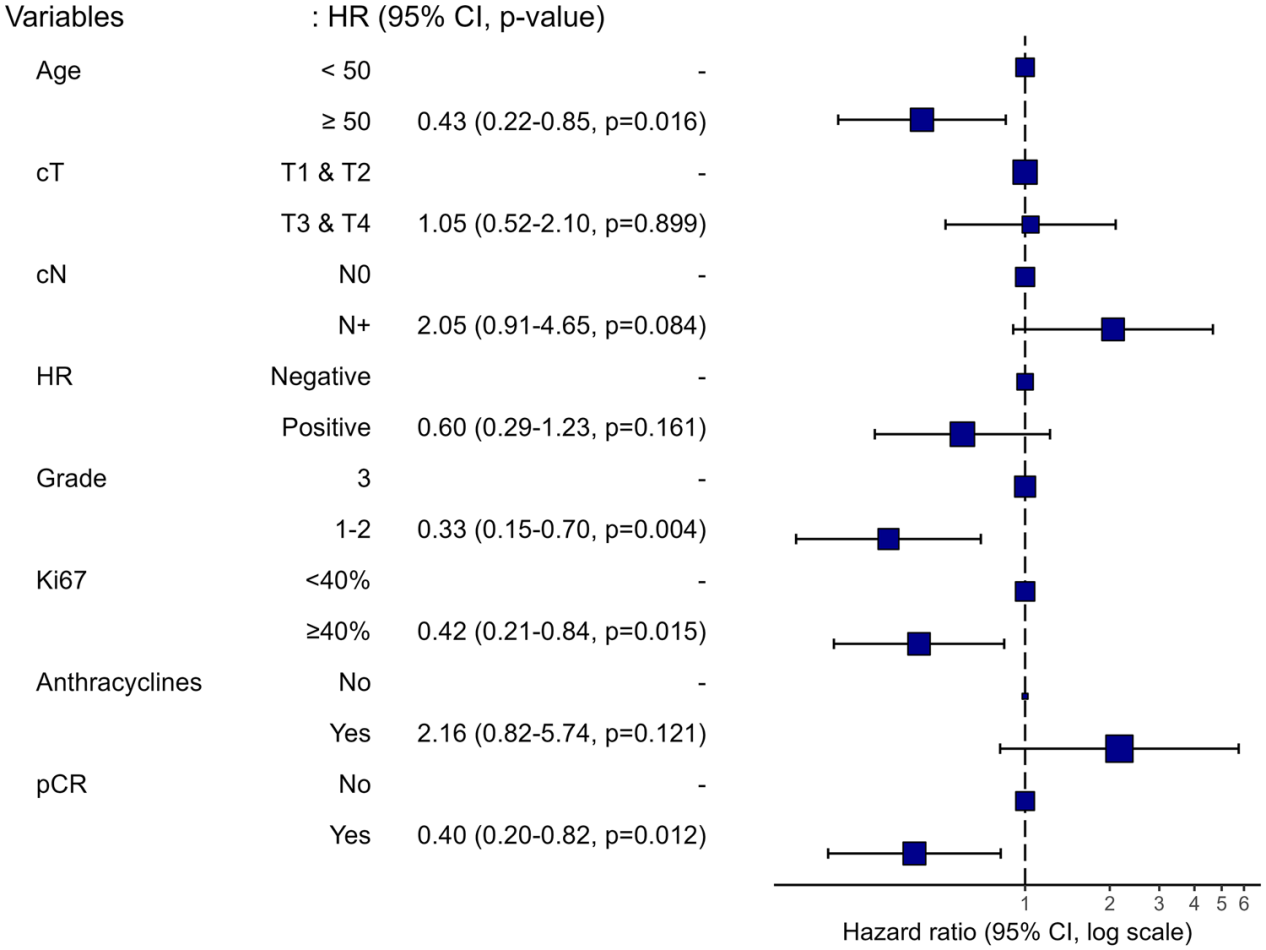
Multivariable Cox regression analysis of factors affecting disease-free survival. cT: tumor size; cN: lymph node disease; HR: hormonal receptor; pCR: pathological complete response

**Fig. 2:**
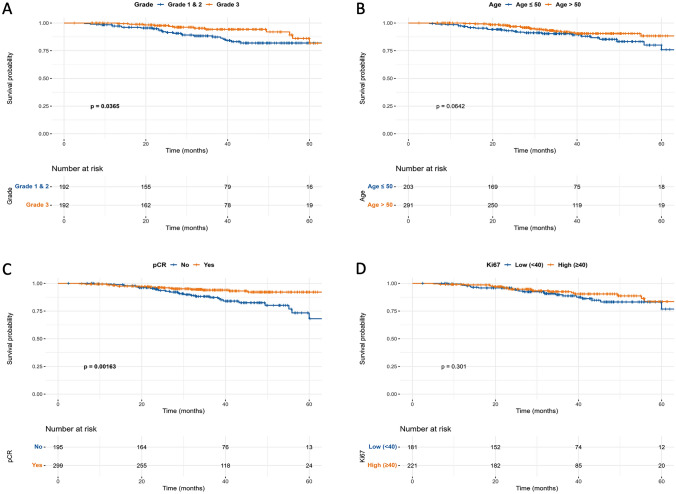
Disease-free survival analysis according to: **A** Grade; **B** age; **C** pathological response; **D** Ki-67 (pCR: pathological complete response). Bold values indicate statistical significance at *p* < 0.05

In the Cox multivariable survival analysis (Fig. 1), age ≥ 50 years, tumor grades 1 or 2, Ki-67 ≥ 40%, and achieving pCR were associated with longer DFS. Figure 2 illustrate the KM curves based on these factors.

## Discussion

Identifying predictive factors for achieving pCR and predicting relapse is crucial in tailoring optimal treatment strategies for patients with HER2+ BC. Real-world data play a significant role in this context due to the inherent heterogeneity not always captured in clinical trials [11].

In our international cohort study, we identified HR expression and nodal status as significant predictive factors for pCR. Age, HR, grade, Ki-67, nodal status, and pathological response were found to be predictive factors for relapse. These findings highlight the importance of comprehensive patient evaluation and the consideration of multiple factors in treatment decision-making for patients with HER2+ BC.

In our analysis, age did not impact pathological response, but it was related to a higher likelihood of relapse. These results are in line with previous literature. In a cohort of 502 patients who received neoadjuvant treatment, age had no impact on pCR, downstaging of the primary tumor, or the lymph nodes [12]. On the other hand, in a review, age < 50 was associated with higher relapse rates; most of the included studies included trastuzumab as the only anti-HER2 therapy [13]. Our findings demonstrate that even with the use of neoadjuvant pertuzumab and adjuvant T-DM1 for residual disease, younger age remains a factor for a higher risk of relapse. This could be explained by the aggressive tumor biology often seen in younger patients [14], despite our analysis not accounting for this factor. Therefore, young age should be considered when evaluating the need for escalating therapy, as it remains a key factor for poor prognosis.

Larger tumor sizes were linked to a higher chance of relapse in the univariate analysis. Other studies have also shown that larger tumors are associated with a lower likelihood of achieving a pCR and a higher risk of relapse [4, 15, 16]. Also, nodal status remains an important factor across all subtypes of BC. Our cohort showed worse responses and higher chances of relapse in patients with nodal-positive disease at diagnosis. Some studies have demonstrated that even in patients who achieve pCR, nodal positivity remains a poor prognostic factor [4, 13, 16, 17]. De-escalating treatment in these patients based on pCR should be done with caution.

A main finding of this study is that patients with HR-positive BC exhibit lower rates of achieving pCR compared to HR-negative patients. A retrospective real-world evidence study conducted using data from the US Oncology Network assessed 238 patients who received neoadjuvant trastuzumab and pertuzumab, achieved pCR, and subsequently received adjuvant trastuzumab. HR– patients were more likely to achieve pCR following NAT. Despite this, the correlation between pCR and favorable long-term outcomes was weaker in the HER2+/HR+ subgroup, potentially due to the additional benefit of adjuvant endocrine therapy [18]. Moreover, relapses in patients with HER2+/HR+ BC tend to occur later and are more likely to involve the bones, compared to relapses in HR–/HER2+ BC patients [2, 19]. A longer follow-up would enable the detection of differences in relapse rates based on HR expression.

Ki-67 remains a controversial biomarker in HER2+ BC due to variability in its interpretation and inconsistencies in the cutoff values used to define high or low expression [20]. Despite these challenges, Ki-67 is a classical factor routinely used in clinical practice to guide treatment decisions. For this reason, we included it in our multivariable analysis. In our cohort, the median Ki-67 value was 40%, which aligns with the literature indicating higher expression in HER2+ tumors [21]. While Ki-67 expression did not significantly impact pCR, Ki-67 levels greater than 40% were associated with a reduced risk of relapse in the multivariate analysis. This finding aligns with published results from a review indicating that baseline Ki-67 expression below 30% consistently correlates with an increased risk of HER2+ recurrence, while higher baseline Ki-67 expression is associated with a reduced recurrence risk of HER2+ BC across various treatment settings [13]. Additionally, a real-world study in China involving 736 patients receiving NAT (with 49% not receiving trastuzumab) found that a Ki-67 level above 30% was a predictor of pCR [22]. Generally, tumors with high Ki-67 expression respond better to treatment but have a poorer prognosis. However, this pattern does not hold for HER2+ tumors, where high Ki-67 levels related to pCR may contribute to a better prognosis.

While tumor grade is generally linked to a higher chance of pCR [21], we did not find any association between grade and pathological response. However, in the multivariable analysis and the Cox regression model, grade 1–2 tumors were associated with a lower probability of relapse, likely related to more favorable tumor biology. Also, we found that omitting anthracyclines did not affect pathological response or DFS, aligning with recent real-world studies and clinical trials [23–26]. This indicates a shift in standard recommendations regarding the use of anthracyclines in the neoadjuvant setting for HER2+ BC. Specifically, the TRAIN-2 and TRYPHAENA trials have shown that anthracycline-free regimens such as TCHP are non-inferior to those containing anthracyclines in terms of pCR and DFS, while also demonstrating lower toxicity [8, 25], However, questions remain regarding the efficacy of anthracycline omission in specific subgroups, such as patients with inflammatory tumors or those with N2 or N3 disease at diagnosis. It's important to note that the present study did not address these subgroups, leaving room for further investigation in these areas.

Achieving a pCR is linked to a better prognosis, as demonstrated by both univariate and multivariate analyses. Extensive literature supports this finding, indicating that pCR is an independent prognostic factor regardless of staging or HR expression. A review found that pCR was the risk factor most closely associated with a decreased risk of relapse [13]. Our research further supports the previous observation that this association is weaker in HR+ tumors (Fig. 2D).

The observed differences in the significance of certain clinicopathological factors between univariate and multivariate models highlight the complexity of interpreting predictors in the context of HER2+ BC outcomes. For instance, Ki-67 did not show statistical significance as a predictor of pCR in either model, suggesting it may not exert a strong independent effect on pCR when other factors, such as HR status and nodal involvement, are considered. However, in the context of relapse, Ki-67 emerged as an independent predictor in the multivariate model, indicating its predictive value may be context-dependent, potentially interacting with other variables like HR status and tumor grade. HR status followed a similar pattern, gaining significance in predicting relapse in the multivariate model. Similarly, tumor grade, which was not significant for predicting pCR, became a significant predictor of relapse in the multivariate model, reflecting its critical role in long-term disease progression when considered alongside other clinicopathological factors. Nodal status also demonstrated significance for predicting pCR only in the multivariate analysis, likely due to the adjustment for confounders that mask its effect in univariate testing. The application of multivariate modeling strengthens our analysis by identifying independent predictors of treatment outcomes, while accounting for confounders and interrelationships among variables. This approach provides a comprehensive understanding of clinicopathological predictors and their context-dependent roles, enhancing the relevance of our findings to real-world clinical scenarios.

Our study benefits from the inclusion of international data, incorporating countries with similar treatment protocols yet diverse population genetic profiles. Despite some variations in treatment approaches across countries, these differences did not have a measurable impact on the primary outcomes of the study—pCR and relapse rates. The broad scope of our study enhances the generalizability of the findings across varied healthcare settings. Additionally, the use of real-world data is a significant strength, as it captures the heterogeneity often overlooked in clinical trials, offering valuable insights into treatment effectiveness in routine clinical practice.

However, our research has limitations worth noting. Firstly, its retrospective nature introduces inherent biases and limitations in data collection, including missing information. Future prospective studies could offer more robust evidence. Furthermore, the relatively short follow-up duration poses a limitation, particularly in assessing differences in relapse. Longer observation periods would allow for a more comprehensive understanding of treatment outcomes over time. Methodological constraints, such as selection bias and incomplete data, may have influenced our results. The choice of the variables was based on their importance in clinical practice for treatment decision-making and characterization of the breast tumor. However, we acknowledge that the lack of adjustments for potential confounders (e.g., country, socioeconomic factors, comorbidities, or treatment adherence) affects the robustness of the results. Implementing strategies to overcome these limitations will strengthen the validity and reliability of future studies.

Additionally, incorporating genomic data and immune profiling, such as tumor-infiltrating lymphocytes (TILs), into these predictive models could provide a more comprehensive understanding of tumor biology [27]. The HER2Dx test, which combines genomic, immune, and clinical data, is an example of a tool that can predict pCR and relapse more accurately [28]. Expanding the use of such tests in research and clinical practice could lead to significant improvements in patient stratification and treatment personalization.

## Conclusion

Real-world studies are important to complement the information from clinical trials and provide a more comprehensive understanding of treatment outcomes. Our findings highlight the multifaceted nature of determining optimal treatment plans for patients. While achieving pCR is a crucial marker of treatment success, our research underscores that it should not be the sole factor guiding therapeutic decisions. Instead, a comprehensive approach that considers age, HR status, and nodal involvement is essential for personalized treatment strategies. These factors should be taken into account when deciding whether to escalate or de-escalate treatment.

## Supplementary Information

Below is the link to the electronic supplementary material.

Supplementary file1 (DOC 179 KB)

## Data Availability

The data that support the findings of this study are available from the corresponding author upon reasonable request.

## Abbreviations

AI: Aromatase inhibitors
BC: Breast cancer
BCS: Breast-conserving surgery
CI: Confidence intervals
cN: Clinical nodal status
cT: Tumor size
DFS: Disease-free survival
HER2+: HER2-positive
HR: Hormonal receptor
IQRs: Interquartile ranges
N+: Positive lymph node involvement
N0: Negative lymph node involvement
NAT: Neoadjuvant therapy
OFS: Ovarian function suppression
pCR: Pathological complete response
TCHP: Carboplatin, docetaxel, trastuzumab and pertuzumab
T-DM1: Trastuzumab emtansine
