# Supplementary material for: Predictive factors for pCR and relapse following neoadjuvant dual HER2-blockade in HER2+ breast cancer: an international cohort study
Source: Clin Transl Oncol. 2025 May 10;27(11):4160–9. doi: 10.1007/s12094-025-03937-7 (PMC12559113; doi:10.1007/s12094-025-03937-7)
Supplement: Supplementary file 1 — Supplementary file1 (DOC 179 KB) [file 12094_2025_3937_MOESM1_ESM.doc]

***Predictive Factors for pCR and Relapse Following Neoadjuvant Dual HER2-Blockade in HER2+ Breast Cancer: An International Cohort Study***

**Supplementary data**

**Supplementary Table 1.** Description analysis of adjuvant anti-HER2 treatment received according to the neoadjuvant regimen treatment

| **Neoadjuvant anthracyclin regimen** | **Adjuvant anti-HER2 treatment** | **Number of patients (%)** |
| --- | --- | --- |
| **No** | None | 1 (0.2) |
|  | Trastuzumab | 134 (25.9) |
|  | T-DM1 | 28 (5.4) |
|  | Trastuzumab+Pertuzumab | 0 |
| **Yes** | None | 3 (0.6) |
|  | Trastuzumab | 301 (58.2) |
|  | T-DM1 | 37 (7.2) |
|  | Trastuzumab+Pertuzumab | 13 (2.5) |

*T-DM1: trastuzumab emtansine.*

**Supplementary Table 2.** Patient characteristics according to pathologic response after neoadjuvant treatment.

| **Patient Characteristics** | **pCR, n (%)** | **non-pCR, n (%)** | **p-value** |
| --- | --- | --- | --- |
| **Total (N = 517)** | 315 (60.9) | 202 (39.1) |  |
| **Age** |  |  |  |
| **< 50 years old** | 130 (41.3) | 83 (41.1) | 0.968 |
| **≥ 50 years old** | 185 (58.7) | 119 (58.9) |  |
| **Menopausal Status** |  |  |  |
| **Pre-menopausal** | 104 (41.1) | 69 (40.1) | 0.920 |
| **Peri/Post-menopausal** | 149 (58.9) | 103 (59.9) |  |
| **HR** |  |  |  |
| **Negative** | 136 (43.2) | 45 (22.3) | **<0.001** |
| **Positive** | 179 (56.8) | 157 (77.7) |  |
| **Ki67 %** |  |  |  |
| **< 40%** | 110 (44.2) | 83 (47.7) | 0.489 |
| **≥ 40%** | 139 (55.8) | 91 (52.3) |  |
| **Histologic Grade** |  |  |  |
| **Grade 1-2** | 118 (49.0) | 90 (54.2) | 0.314 |
| **Grade 3** | 123 (51.0) | 76 (45.8) |  |
| **cT** |  |  |  |
| **T1-2** | 211 (67.0) | 133 (65.8) | 0.849 |
| **T3-4** | 104 (33.0) | 69 (34.2) |  |
| **cN** |  |  |  |
| **N0** | 143 (45.4) | 78 (38.6) | 0.145 |
| **N+** | 172 (54.6) | 124 (61.4) |  |
| **Neoadjuvant Chemotherapy** |  |  |  |
| **Anthracycline based** | 212 (67.3) | 142 (70.3) | 0.498 |
| **TCHP** | 103 (32.7) | 60 (29.7) |  |
| **Adjuvant Anti-HER2** |  |  |  |
| **Trastuzumab** | 306 (97.1) | 129 (63.9) | **<0.001** |
| **Trastuzumab + Pertuzumab** | 6 (1.9) | 7 (3.5) |  |
| **T-DM1** | 3 (1.0) | 62 (30.7) |  |
| **None** | 0 (0.0) | 4 (2.0) |  |
| **Adjuvant Endocrine Therapy** |  |  |  |
| **Tamoxifen** | 37 (20.7) | 31 (19.7) | 0.756 |
| **AI** | 87 (48.6) | 80 (51) |  |
| **Tamoxifen + OFS** | 18 (10.1) | 12 (7.6) |  |
| **AI + OFS** | 14 (7.8) | 12 (7.6) |  |
| **Unknown** | 23 (12.8) | 22 (14.0) |  |
| **Type of Primary Surgery** |  |  |  |
| **Mastectomy** | 159 (50.5) | 115 (56.9) | 0.123 |
| **BCS** | 156 (49.5) | 87 (43.1) |  |
| **Type of Axillary Surgery** |  |  |  |
| **Complete Axillary Dissection** | 146 (49.5) | 122 (60.4) | **0.004** |
| **SLN** | 163 (51.7) | 79 (39.1) |  |
| **None** | 6 (1.9) | 1 (0.5) |  |

*AI: aromatose inhibitors; BCS: breast conservative surgery; cT: tumor size; cN: Lymph node disease; HR: hormonal receptor; OFS: Ovary function suppression; pCR: pathologic complete response; TCHP: Docetaxel, carboplatin, trastuzumab and pertuzumab; SLN: Sentinel lymph node biopsy.*

**Supplementary Table 3.** Patient characteristics according to the occurrence of relapse during follow-up.

| **Patient characteristics** | **Relapse, n (%)** | **non-Relapse, n (%)** | **p-value** |
| --- | --- | --- | --- |
| **Total (N=517)** | 39 (7.5) | 478 (92.5) |  |
| **Age** |  |  |  |
| **< 50 years old** | 23 (59.0) | 190 (39.7) | **0.027** |
| **≥ 50 years old** | 16 (41.0) | 288 (60.3) |  |
| **Menopausal Status** | 35 (100) | 390 (100) |  |
| **Pre-menopausal** | 15 (42.9) | 158 (40.5) | 0.858 |
| **Peri/Post-menopausal** | 20 (57.1) | 232 (59.5) |  |
| **HR** |  |  |  |
| **Negative** | 17 (43.6) | 164 (34.3) | 0.295 |
| **Positive** | 22 (56.4) | 314 (65.7) |  |
| **Ki67 %** | 34 (100) | 389 (100) |  |
| **< 40%** | 19 (55.9) | 174 (44.7) | 0.216 |
| **≥ 40%** | 15 (44.1) | 215 (55.3) |  |
| **Histologic grade** | 32 (100) | 375 (100) |  |
| **Grade 1-2** | 11 (34.4) | 197 (52.5) | 0.053 |
| **Grade 3** | 21 (65.6) | 178 (47.5) |  |
| **cT** |  |  |  |
| **T1-2** | 19 (48.7) | 325 (68) | **0.021** |
| **T3-4** | 20 (51.3) | 153 (32) |  |
| **cN** |  |  |  |
| **N0** | 8 (20.5) | 213 (44.6) | **0.005** |
| **N+** | 31 (79.5) | 265 (55.4) |  |
| **Neoadjuvant Chemotherapy** |  |  |  |
| **Anthracycline based** | 30 (76.9) | 324 (67.8) | 0.284 |
| **TCHP** | 9 (23.1) | 154 (32.2) |  |
| **Adjuvant anti-HER2** |  |  |  |
| **Trastuzumab** | 31 (79.5) | 404 (84.5) | 0.083 |
| **Trastuzumab+Pertuzumab** | 3 (7.7) | 10 (2.1) |  |
| **T-DM1** | 4 (10.3) | 61 (12.8) |  |
| **None** | 1 (2.6) | 3 (0.6) |  |
| **Adjuvant endocrine therapy** |  |  |  |
| **Tamoxifen** | 9 (40.9) | 60 (21.4) | 0.157 |
| **AI** | 8 (36.4) | 162 (57.7) |  |
| **Tamoxifen+OFS** | 2 (9.1) | 28 (10) |  |
| **AI+OFS** | 3 (13.6) | 23 (8.2) |  |
| **Unknown** | 0 (0.0) | 8 (2.8) |  |
| **Type of primary surgery** |  |  |  |
| **Mastectomy** | 26 (66.7) | 248 (51.9) | 0.063 |
| **BCS** | 13 (33.3) | 230 (48.1) |  |
| **Type of axillary surgery** |  |  |  |
| **Complete axillary dissection** | 29 (74.4) | 239 (50.0) | **0.007** |
| **SLN** | 9 (23.1) | 232 (48.5) |  |
| **None** | 0 (0.0) | 7 (1.4) |  |
| **Pathological response** |  |  |  |
| **pCR** | 14 (35.9) | 301 (63.0) | **0.001** |
| **non-pCR** | 25 (64.1) | 177 (37.0) |  |
| **Local of relapse**  **Locorregional** | 3 (7.7) |  |  |
| **New BC** | 1 (2.6) |  |  |
| **Distant** | 35 (89.7) |  |  |
| **Brain** | 18 (51.4) |  |  |
| **Bone** | 5 (14.3) |  |  |
| **Nodal** | 5 (14.3) |  |  |
| **Lung** | 3 (8.6) |  |  |
| **Liver** | 2 (5.7) |  |  |
| **Skin** | 2 (5.7) |  |  |
|  |  |  |  |

*AI: aromatose inhibitors; BC: breast cancer; BCS: breast conservative surgery; cT: tumor size; cN: Lymph node disease; HR: hormonal receptor; OFS: Ovary function suppression; pCR: pathologic complete response; TCHP: Docetaxel, carboplatin, trastuzumab and pertuzumab; SLN: Sentinel lymph node biopsy.*
